# Case report and literature review: clinical manifestations and treatment of human RelA deficiency

**DOI:** 10.3389/fimmu.2025.1529654

**Published:** 2025-02-27

**Authors:** Chenghao Wang, Wenjie Wang, Xiaoying Hui, Jia Hou, Qinhua Zhou, Qifan Li, Qi Wu, Qi Ni, Bingbing Wu, Jinqiao Sun, Xiaochuan Wang

**Affiliations:** ^1^ Department of Clinical Immunology, National Children Medical Center, Children’s Hospital of Fudan University, Shanghai, China; ^2^ Clinical Genetic Center, National Children Medical Center, Children’s Hospital of Fudan University, Shanghai, China; ^3^ Shanghai Institute of Infectious Disease and Biosecurity, Shanghai, China

**Keywords:** RelA deficiency, NF-κB, recurrent oral ulcers, Behçet’s disease, case report

## Abstract

RelA deficiency resulting from mutations in the human *RELA* gene is a recently identified inborn errors of immunity (IEI). The *RELA* gene encodes the RelA (p65) protein, one of the five transcription factors of the NF-κB family, which plays a critical role in the regulation of transcriptional programs essential for the development and maintenance of the immune system, skeletal system, and epithelial tissues. RelA deficiency is classified as RelA haploinsufficiency and RelA dominant-negative. The mainly pathogenesis is that impaired NF-κB activation in fibroblasts, which leads to the downregulation of NF-κB-dependent antiapoptotic protein expression and cytokine transcription, renders fibroblasts susceptible to TNF-induced apoptosis. Clinical manifestations of RelA deficiency are typically characterized by recurrent oral ulcers or Behçet’s disease-like manifestations. Since the first report in 2016, only a few dozen cases of RelA deficiency have been documented worldwide. Treatment strategies have not been standardized, with current mainstream approaches primarily involving immunosuppressive therapies, including TNF inhibitors or glucocorticoids. In this study, we report the clinical phenotypes of three patients with RelA deficiency from two families, along with one novel pathogenic mutation (c.1166_1184del, p.Q389fs) in the *RELA* gene. This expands the spectrum of pathogenic mutations associated with the *RELA* gene and clinical manifestations of RelA deficiency. Additionally, we provide a comprehensive summary of the genetic phenotypes, clinical characteristics, and treatment strategies of all previously reported cases of RelA deficiency. Our aim is to increase awareness of this rare IEI and to offer insights that may guide its treatment.

## 1 Introduction

NF-κB is a class of transcriptional regulators comprising five family members: RelA (p65), RelB, c-Rel, NF-κB1 (p50), and NF-κB2 (p52). These proteins typically exist as homodimers or heterodimers in the cytoplasm and can be activated by a variety of membrane-bound and soluble extracellular ligands, particularly members of the TNFR, TLR, IL-1R, and antigen receptor superfamilies. Upon activation, NF-κB dimers translocate to the nucleus, where they bind to κB sites on DNA to regulate transcription (1). The target genes regulated by NF-κB can be categorized into four main functional groups: inflammatory and immunoregulatory genes, antiapoptotic genes, genes that positively regulate the cell cycle, and genes that encode negative regulators of NF-κB (2). The transcriptional programs regulated by NF-κB are critical for the development and maintenance of the immune system, skeletal system, and epithelial tissues. In these processes, the NF-κB pathway plays a pivotal role in controlling cell survival, differentiation, and proliferation (1).

Deficiencies in any of the NF-κB family subunits can impair NF-κB function, leading to inborn errors of immunity (IEI), which have been increasingly recognized over the past decade. Among these, NFKB2 deficiency and NFKB1 deficiency, which were first reported in 2013 and 2015, respectively, are autosomal dominant forms of common variable immune deficiency (CVID) (3, 4). RelB deficiency and c-Rel deficiency, reported for the first time in 2015 and 2019, respectively, are autosomal recessive forms of combined immunodeficiency (CID) (5–8).

The *RELA* gene is located on chromosome 11q13.1 (9) and encodes the RelA (p65) protein, which is structurally composed of an N-terminal Rel homology domain (RHD) and a C-terminal transactivation domain (TAD). The RHD makes contact with DNA and supports subunit dimerization, while the TAD confers the ability to initiate transcription (1). The functions of RelA include maintaining normal embryonic development, regulating lymphocyte development and proliferation, producing specific immunoglobulin isotypes, and ensuring regulatory T-cell populations (10).

RelA deficiency was first reported in 2016 as an autosomal dominant disorder. To date, only a few dozen cases of RelA deficiency have been documented worldwide. Unlike deficiencies in other NF-κB family subunits, RelA deficiency minimally affects lymphocyte function. Depending on the pathogenesis, RelA deficiency is classified as RelA haploinsufficiency and RelA dominant-negative (11, 12). The common pathogenesis is that impaired NF-κB activation in fibroblasts, which leads to the downregulation of NF-κB-dependent antiapoptotic proteins and cytokines, increases the susceptibility of fibroblasts to TNF-induced apoptosis. Consequently, patients with RelA deficiency typically present with recurrent oral ulcers or Behçet’s disease-like symptoms, with few cases of recurrent infections. Unlike RelA haploinsufficiency, RelA dominant-negative additionally leads to excessive interferon (IFN) production, which result in type I interferonopathy-like clinical manifestations. Given the limited number of reported cases, treatment strategies for RelA deficiency have not yet been standardized. Immunosuppressive therapies, primarily TNF inhibitors or glucocorticoids, constitute the current mainstream approach.

In this study, we report the clinical phenotypes of three patients with RelA deficiency from two families, alongside one novel pathogenic mutation in the *RELA* gene. (This study was approved by the Ethics Committee of the Children’s Hospital of Fudan University No. 2022 100. All patients’ guardians provided written informed consent for enrollment in this study.) These findings expand the known spectrum of pathogenic mutations in the *RELA* gene and clinical manifestations of RelA deficiency. Additionally, we provide a comprehensive summary of the genetic phenotypes, clinical characteristics, and treatment strategies of all previously reported cases of RelA deficiency. Our goal is to increase awareness of this rare IEI and to provide insights that may guide its treatment.

## 2 Case description

### 2.1 Clinical manifestations

**Figure 1:**
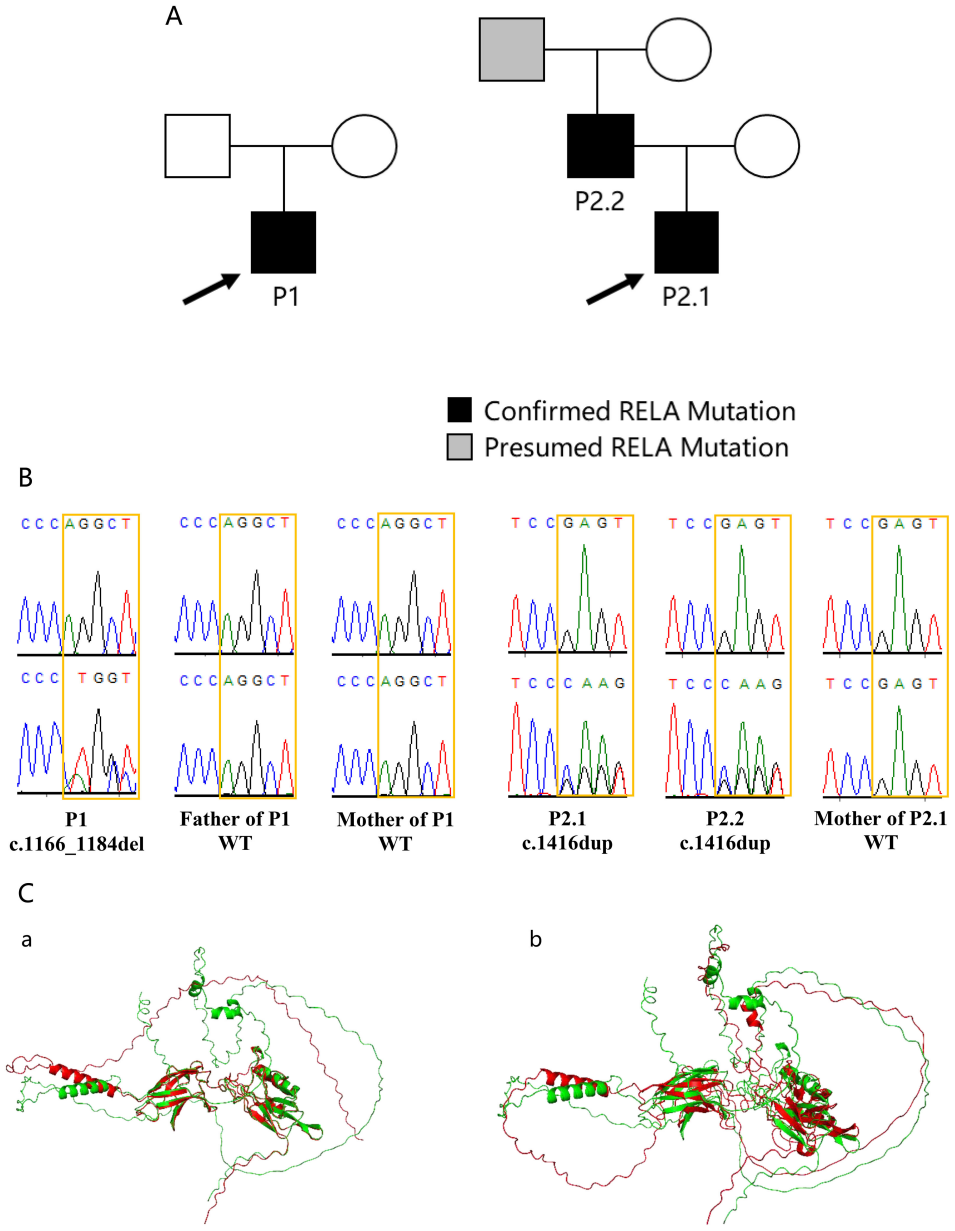
Characteristics of *RELA* gene mutations in patients with RelA deficiency. **(A)**: Pedigrees of patients with RelA deficiency and their families. **(B)**: Sanger sequencing of the *RELA* mutation. **(C)**: Structural comparison of mutant and wild-type RelA (p65) proteins (13). The overlapping colors represented the same three-dimensional structure, and the non-overlapping colors represented different three-dimensional structures. a: The red structure represents the p.Q389fs mutant and the green structure represents the wild-type protein. b: The red structure represents the p.E473fs mutant and the green structure represents the wild-type protein.

P1 (**Figure 1A**), a 4-year-old boy, began experiencing recurrent oral ulcers at the age of 2. Each episode lasted approximately 15 days, with a 5- to 6-day remission before recurrence. These episodes were accompanied by recurrent fevers, during which the tonsils were swollen and erythematous. The fevers typically resolved within 2~3 days following the administration of antipyretics and antibiotics, with intervals of 7~30 days between fever episodes. Notably, there was little evidence of infection during these episodes. The patient did not present with rashes, abdominal pain, diarrhea, or joint discomfort during the febrile periods. In addition, the patient had no chronic lymphadenopathy or splenomegaly. Laboratory tests during febrile episodes revealed fluctuating peripheral white blood cell counts between 6~13 ×10^9^/L, with neutrophil percentages ranging from 35% to 60%. C-reactive protein (CRP) levels fluctuated between 10~30 mg/L, while the levels of various cytokines, such as IL-1, IL-6, IFN-α, IFN-γ and TNF-α, remained within the normal range. There was no family history of recurrent oral ulcers. Because of normal gastrointestinal endoscopy results, the patient’s family declined the use of immunomodulatory therapies, and no further follow-up was conducted.

P2.1 (**Figure 1A**), an 11-year-old girl, began experiencing recurrent oral ulcers at the age of 5, followed by recurrent diarrhea starting at the age of 9. She experienced 3~8 bowel movements per day, characterized by loose stools without mucus or blood, accompanied by intermittent periumbilical abdominal pain that was unrelated to food intake and could resolve spontaneously. The patient suffered from severe malnutrition but did not experience recurrent fevers or rashes. In addition, the patient had no chronic lymphadenopathy or splenomegaly. Gastrointestinal endoscopy revealed rough gastric fundus mucosa, congested and edematous mucosa in the gastric body, an abundance of mucus, rough antral mucosa, congested and edematous mucosa in the gastric antrum, and congested and edematous mucosa with visible erythema and partial surface erosion in the duodenal bulb and postbulbar region. The terminal ileum showed congested and edematous villi with erosion and mucus presence, while the mucosa of the sigmoid colon and rectum was congested and edematous with visible erythema. Histopathological examination revealed mild chronic superficial gastritis in the gastric antrum (*Hp*-negative), focal gastric mucosal metaplasia in the duodenal bulb, and increased eosinophilic infiltration in the stroma (70~90 cells/HPF). Additionally, focal eosinophilic infiltration (20 cells/HPF) was observed in the small intestine. Peripheral blood tests revealed a normal total white blood cell count with eosinophilia (1,700 cells/μL). The levels of inflammatory markers, including CRP and various cytokines, such as IL-1, IL-6, IFN-α, IFN-γ and TNF-α, were within normal ranges. Treatment with glucocorticoids significantly alleviated the diarrhea, but symptoms recurred upon discontinuation of the medication. The patient is currently maintained on a low-dose glucocorticoid regimen. The patient’s father (P2.2) and grandfather both had a history of recurrent oral ulcers since childhood, but neither had received immunomodulatory treatment.

### 2.2 Immunological features

The immunological profile of the patients is summarized in **Table 1**. P1: The proportions and counts of T, B, and NK cells were within normal ranges. There was a reduction in the proportion of CD4+ central memory T cells (TCM), with an increase in the proportions of effector memory T cells (TEM) and terminal effector T cells (TTE). IgA levels were elevated, whereas IgM levels were decreased. P2.1: This patient exhibited a marked increase in NK cell count and a reduction in CD4+ T-cell numbers. Similar to P1, there was a decrease in CD4+ TCM and an increase in TEM and TTE. Additionally, there was a decrease in CD8+ TCM and an increase in TTE. The B-cell counts were normal, but the IgG and IgA levels were slightly above the upper normal limits, the IgM level was slightly below the lower normal limit, and the IgE level was significantly elevated. Further testing revealed elevated levels of specific IgE against multiple food allergens, including nuts, egg whites, wheat, sesame, peanuts, and shrimp. No positive autoantibody, such as antinuclear antibody (ANA), extractable nuclear antigen antibody (ENA) and anti-neutrophil cytoplasmic antibody (ANCA), was found in P1 and P2.1. P2.2: Routine immune function testing was not performed.

**Table 1:** Lymphocyte subsets and immunoglobulin levels in two patients with RelA deficiency.

	P1	Reference Range	P2.1	Reference Range
CD3+ (%)	67	64-73	41	64-73
CD3+ (×10^6^/ml)	2559	1410-3380	1368	1410-3380
CD4+ (%)	37	29-36	17	29-36
CD4+ (×10^6^/ml)	1419	710-1840	567	710-1840
% in CD4+				
CD27+CD45RA+	65	46-75	50	40-72
CD27+CD45RA-	17	22-46	21	23-51
CD27-CD45RA-	11	2-9	24	3-10
CD27-CD45RA+	7	0-1	5	0-2
CD8+ (%)	22	24-34	20	24-34
CD8+ (×10^6^/ml)	843	540-1360	664	540-1360
% in CD8+				
CD27+CD45RA+	56	42-78	57	36-72
CD27+CD45RA-	24	12-31	9	13-39
CD27-CD45RA-	5	2-13	2	2-17
CD27-CD45RA+	15	2-25	33	1-22
CD19+ (%)	15	14-21	14	14-21
CD19+ (×10^6^/ml)	594	450-820	481	450-820
CD16+CD56+ (%)	17	11-23	44	11-23
CD16+CD56+ (×10^6^/ml)	662	280-630	1487	280-630
IgG (g/L)	9.40	4.95-12.74	16.60	6.09-12.85
IgA (g/L)	2.13	0.33-1.89	3.04	0.52-2.16
IgM (g/L)	0.59	0.65-2.01	0.55	0.67-2.01
IgE (KU/L)	48.38	<100	907.74	<100

CD27+CD45RA+: Naïve T cells; CD27+CD45RA-: central memory T cells (TCM); CD27-CD45RA-: effector memory T cells (TEM); CD27-CD45RA+: terminal effector T cells (TTE).

### 2.3 Genetic characteristics

Whole-exome sequencing (WES) and Sanger sequencing (14) were adapted for identifying genetic variants in P1, P2.1, and their parents. Apart from mutations in the *RELA* gene, no other pathogenic gene mutations were identified. P1: A *de novo* heterozygous mutation in the *RELA* gene (c.1166_1184del, p.Q389fs) was detected (**Figure 1B**). P2.1: A heterozygous mutation in the *RELA* gene (c.1416dup, p.E473fs), which was inherited from the father (P2.2), was identified. The mother tested negative for *RELA* gene mutations (**Figure 1B**). Unfortunately, blood samples from P2.1’s paternal grandparents were not available for analysis.

### 2.4 Pathogenicity analysis

The *RELA* gene mutation c.1166_1184del (p.Q389fs) identified in this study is a novel variant that has not been previously reported in the Human Gene Mutation Database (HGMD) or the Genome Aggregation Database (gnomAD) (ALL: 0.000%). This mutation leads to premature termination of amino acid translation, and according to the American College of Medical Genetics and Genomics (ACMG) variant classification guidelines, this is classified as “likely pathogenic” variant. We employed AlphaFold 3 for protein structure prediction (15), structural prediction analysis of the mutant protein revealed that this mutation not only truncates the RelA (p65) protein but also causes significant alterations in its three-dimensional structure (**Figure 1C**). Furthermore, the length of the protein encoded by this mutation is shorter than those of the previously confirmed pathogenic variant p.H487fs (16). Additionally, the carrier of this novel *RELA* gene mutation exhibits the typical clinical manifestations of RelA deficiency. Taken together, these findings provide strong evidence supporting the pathogenicity of the *RELA* gene mutation c.1166_1184del (p.Q389fs).

Previous studies have confirmed that the mutation c.1416dup (p.E473fs) (gnomAD: ALL: 0.000%) truncates RelA protein (12), which is consistent with the protein structural prediction in this study (**Figure 1C**). This also confirmed the reliability of the protein structural prediction method in this study.

## 3 Discussion

Literature searches for available data were conducted in the PubMed database (http://www.ncbi.nlm.nih.gov/pubmed/) using the option “Advanced Search”, selecting “All Fields” in the search builder and inputting “RELA” in the search box. The inclusion criteria included case report studies on human *RELA* gene mutation-associated diseases written in English. The exclusion criterion was studies published in languages other than English. Available data, such as abstracts or full-text articles and related citations and references, were reviewed.

**Figure 2:**
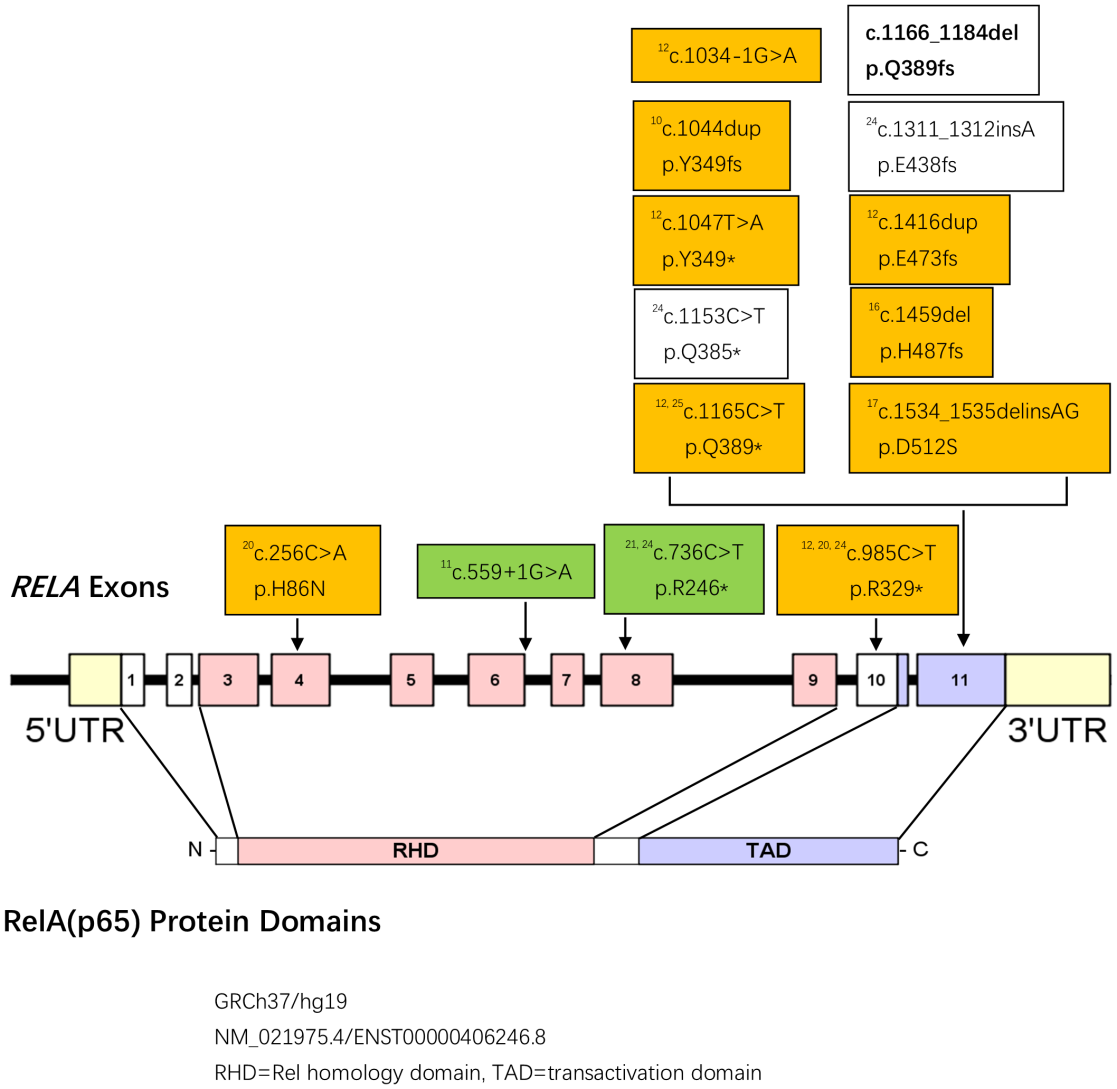
(18, 19) Pathogenic mutations in RelA deficiency (Newly identified mutations in this study are bolded. Superscript numbers correspond to the references reporting each mutation. Dominant-negative mutations are shown in orange frame, while haploinsufficiency mutations are shown in green frame. Effect types of mutations shown in white frame are unknown).

Since the first case reported by Frederiksen et al. (17) in 2016, a total of nine studies meeting the inclusion criteria were published up to June 2024. In this study, we report two pathogenic mutations in the *RELA* gene, including one novel pathogenic mutation, which were identified in three patients from two families. Until now, a total of 14 confirmed pathogenic *RELA* gene mutations (**Figure 2**) across 19 families, encompassing 47 patients, were identified. Detailed information is provided in **Supplementary Table 1**. All of the pathogenic *RELA* gene mutations reported in the literature, including those described in this study, are heterozygous and exhibit autosomal dominant inheritance. The majority of these pathogenic mutations are located in exon 11, corresponding to the TAD of the RelA protein. Of the 14 pathogenic mutations, 9 were confirmed as dominant-negative and 2 were confirmed as haploinsufficiency. The confirmed haploinsufficiency occurred only in the RHD of the RelA protein, whereas the dominant-negative occurred mainly in the TAD but a few could also occur in the RHD. RelA deficiency can manifest at any age, with the onset most commonly occurring during childhood. The median age of onset was 5 (2, 10) years, with the earliest reported onset occurring shortly after birth and the latest occurring at 33 years of age. The clinical manifestations of RelA deficiency are diverse and can affect multiple organ systems (**Table 2**). The most common presentations were recurrent oral ulcers (32%, 15/47), followed by Behçet’s disease-like symptoms (30%, 14/47) and gastrointestinal involvement (30%, 14/47). Additionally, autoimmune diseases such as systemic lupus erythematosus (SLE), immunologic thrombocytopenic purpura, autoimmune neutropenia, autoimmune lymphoproliferative syndrome (ALPS), Sjögren’s syndrome, and neuromyelitis optica-like manifestations have been reported in isolated cases. Behçet’s disease, SLE, autoimmune hematological disease, Sjögren’s syndrome, and neuromyelitis optica only occurred in the dominant-negative, while ALPS and solitary conjunctivitis only occurred in the haploinsufficiency. Specific clinical data for each patient are detailed in **Supplementary Table 1**.

**Table 2:** Summary of clinical manifestations of patients with RelA deficiency.

Clinical manifestation	Number of patients (N=47)	Percentage (%)	Haploinsufficiency	Dominant-negative
Recurrent oral ulcers^1^	15	32	√	√
Behçet’s disease	14	30		√
Gastrointestinal symptoms^2^	14	30	√	√
Musculoskeletal involvement^3^	9	19	√	√
Recurrent fever^4^	7	15	√	√
Recurrent genital ulcers^5^	4	9	√	√
SLE	4	9		√
Dermatic involvement ^6^	4	9	√	√
Recurrent infections^7^	3	6	√	√
Autoimmune hematological disease^8^	1	2		√
ALPS	1	2	√	
Sjögren’s syndrome	1	2		√
Neuromyelitis optica^9^	1	2		√
Conjunctivitis^10^	1	2	√	

1. Patients with Behçet’s disease or SLE were excluded.

2. Includes recurrent abdominal pain, vomiting, diarrhea, and hematochezia. Endoscopic findings include superficial gastritis, duodenitis, ileitis, colitis, cryptitis, eosinophilic gastroenteritis, gastrointestinal mucosal ulcers and inflammatory bowel disease. Patients with Behçet’s disease or SLE were excluded.

3. Includes joint pain, juvenile idiopathic arthritis, high bone mass, muscle weakness, myalgia, and scoliosis. Patients with Behçet’s disease, SLE, or Sjögren’s syndrome were excluded.

4. Patients with infection, Behçet’s disease, SLE, or Sjögren’s syndrome were excluded.

5. Patients with Behçet’s disease or SLE were excluded.

6. Includes allergic-like rash, erythema, papules, vesicles, skin ulcers, and subcutaneous nodules, affecting the trunk, limbs, face, and scalp. Excludes patients with infection, Behçet’s disease, SLE, or Sjögren’s syndrome.

7. Includes pharyngitis, laryngitis, pneumonia, otitis media, conjunctivitis, cellulitis, and generalized bullous lesions. Pathogens include virus, bacterium and fungus.

8. Includes immunologic thrombocytopenic purpura and autoimmune neutropenia.

9. Recurrent optic neuritis, positive for AQP-4 antibodies, without evidence of transverse myelitis or brain involvement.

10. Excludes patients with infection, Behçet’s disease, SLE, or Sjögren’s syndrome, with no other identified cause.

The primary pathogenic mechanism of RelA deficiency involves impaired NF-κB activation in fibroblasts, leading to the downregulation of NF-κB-dependent antiapoptotic proteins and cytokines, which increases the susceptibility of fibroblasts to TNF-induced apoptosis (11). Mucosal tissues in the mouth, gastrointestinal tract, and vagina are rich in microbes and prone to inflammatory stimuli, which frequently induce TNF release. In patients with RelA deficiency, excessive fibroblast apoptosis impairs mucosal repair, resulting in recurrent mucosal ulcers. Given the ubiquitous nature of the NF-κB signaling pathway and its involvement in the regulation of hundreds of target genes, RelA deficiency may also present with autoimmune diseases owing to its potential impact on inflammatory responses, autoimmunity, and carcinogenesis (17).

RelA deficiency is divided into RelA haploinsufficiency and RelA dominant-negative, RelA haploinsufficiency refers to a heterozygous mutation of the *RELA* gene that renders the RelA proteins expressed on one chromosome inactive but do not affect the normal RelA proteins expressed on the other chromosome. Insufficient expression of normal RelA proteins leads to impaired NF-κB activation. RelA dominant-negative refers to the situation that the mutant RelA proteins not only do not function normally, but also form heterodimers with the normal RelA proteins, thereby making the normal RelA proteins function impaired. It has been shown that RelA dominant-negative not only impairs NF-κB activation, but also leads to the excessive production of IFN, which induces type I interferonopathy-like manifestations (12, 20). In addition, Frederiksen et al. (17) also speculated that some RelA dominant-negative would affect osteoblasts differentiation and lead to excessive bone formation.

Among the 11 known pathogenic mutations in the non-RHD segment of RelA protein, 8 have been confirmed to exert a dominant-negative effect, and a missense mutation in the RHD has also been confirmed to exert a dominant-negative effect. This may be because these variants do not affect its ability to form dimers. Therefore, we speculate that the remaining 3 known pathogenic mutations in the TAD also exert a dominant-negative effect, which needs to be confirmed by further experiments. If this can be confirmed, the type of effect of the subsequent newly discovered pathogenic mutations may be predicted to a certain extent.

Mutations at different sites within the *RELA* gene can lead to varying clinical manifestations, and even the same mutation may result in different clinical outcomes. Frederiksen et al. (17) suggested that different mutation sites may influence the expression of distinct NF-κB target genes. Comrie et al. (21) proposed that potentially deleterious variants in genes associated with the NF-κB pathway can lead to varying clinical phenotypes. Adeeb et al. (16) speculated that the differing clinical manifestations of the same mutation might share a similar pathophysiological basis.

Although classified as combined immunodeficiency (22), patients with RelA deficiency typically do not exhibit pronounced susceptibility to infections. Animal studies have demonstrated that lymphocyte function in mice with RelA deficiency is not significantly impaired (23). Previous reports have shown that patients with RelA deficiency do not exhibit significant reductions in lymphocyte counts or immunoglobulin levels; in fact, some patients display elevated levels of memory T cells, terminally differentiated T cells, IgG, and IgE (10, 12, 18, 24), which is consistent with the findings in the patients reported in this study. Further research is needed to elucidate the impact of human RelA deficiency on lymphocyte function.

Owing to the rarity of cases and the variability in clinical presentations, there is currently no consensus on the treatment of RelA deficiency. Existing therapeutic approaches focus primarily on controlling inflammation. Given the established pathogenic role of TNF, TNF inhibitors (including etanercept, infliximab, adalimumab, and golimumab) have been widely used (30%, 14/47). Glucocorticoids, as broad-spectrum anti-inflammatory agents, are also commonly employed (28%, 13/47). Additionally, the use of colchicine, rituximab, IL-1 inhibitors (anakinra, canakinumab), and apremilast (a phosphodiesterase-4 inhibitor) has been reported. As an accepted curative treatment for IEI, hematopoietic stem cell transplantation (HSCT) was successfully used in a patient presenting with chronic immunologic thrombocytopenic purpura, autoimmune neutropenia and inflammatory bowel disease. In addition, Moriya et al. (12) suggested that Janus kinase inhibitors are a promising option for cases refractory to TNF inhibitors or other therapy. The prognosis for patients with RelA deficiency is generally favorable. Except for one case involving a neonate with high bone mass at birth who died shortly after birth (17), all of the other treated patients experienced effective symptom relief. The specific treatments and outcomes for each patient are detailed in **Supplementary Table 1**. Moriya et al. (12) suggested that patients with RelA dominant-negative have severer phenotypes and worse clinical outcomes than those with RelA haploinsufficiency. Although the summary of the clinical manifestations of the patients with RelA deficiency in this study showed that systemic autoinflammation and autoimmunity in RelA dominant-negative were indeed more common than RelA haploinsufficiency. But that is not absolute. A 15-year-old male patient with RelA dominant-negative who presented with recurrent oral ulcers achieved spontaneous remission in adulthood without treatment (16). In contrast, the treatment of a patient with RelA haploinsufficiency presenting with ALPS was not easy. Thus, larger studies with longer follow-up periods are needed to better understand the prognosis of patients with RelA deficiency.

In this study, we report a novel pathogenic mutation in the *RELA* gene. Additionally, we provide the most comprehensive summary of the genetic phenotypes, clinical characteristics, and treatment strategies of all previously reported cases of RelA deficiency. This provides important value for the clinical diagnosis and treatment of RelA deficiency. The main limitation of this study is the lack of functional validation for the new mutations reported here. The pathogenicity analysis was mainly based on protein structure prediction in silico, absence in experimental verification.

In summary, RelA deficiency is a rare IEI characterized by autosomal dominant inheritance. Clinically, it typically presents with recurrent oral ulcers or Behçet’s disease-like manifestations. The mainstay of treatment involves immunosuppressive therapies, with TNF inhibitors and glucocorticoids being the most commonly used. For patients with typical symptoms, early comprehensive genetic testing can facilitate timely intervention and provide valuable genetic counseling.

## Data Availability

The datasets presented in this article are not readily available because of ethical and privacy restrictions. Requests to access the datasets should be directed to the corresponding authors.
