# Supplementary material for: Case report and literature review: clinical manifestations and treatment of human RelA deficiency
Source: Front Immunol. 2025 Feb 27;16:1529654. doi: 10.3389/fimmu.2025.1529654 (PMC11934250; doi:10.3389/fimmu.2025.1529654)
Supplement: Supplementary file 1 [file Table1.docx]

**Supplementary Table 1** Detailed clinical data of patients with RelA deficiency.

|  | Sex | Age at Onset (Years) | Main Clinical Manifestations | Effective Treatment | Outcome | Mutation Site^*^ | Effect Type | Reference |
| --- | --- | --- | --- | --- | --- | --- | --- | --- |
| P1 | Male | 2 | Recurrent oral ulcers, recurrent fever | - | - | c.1166_1184del  p.Q389fs | Unknown | This study |
| P2.1 | Female | 5 | Recurrent oral ulcers, eosinophilic gastroenteritis | Glucocorticoids | Improved | c.1416dup  p.E473fs | Dominant-negative | This study |
| P2.2 | Male | Childhood | Recurrent oral ulcers | - | - | c.1416dup  p.E473fs | Dominant-negative | This study |
| P3 | Male | 0 (Newborn) | High bone mass | - | Deceased | c.1534_1535delinsAG  p.D512S | Dominant-negative | FREDERIKSEN A L, et al. 2016(1) |
| P4.1 | Female | 3 | Recurrent fever, recurrent oral ulcers, ileitis | Glucocorticoids, infliximab, methotrexate | Improved | c.559+1G>A  splicing variants | Haploinsufficiency | BADRAN Y R, et al. 2017(2) |
| P4.2 | Female | 2 | Recurrent oral and genital ulcers | Glucocorticoids | Improved | c.559+1G>A  splicing variants | Haploinsufficiency | BADRAN Y R, et al. 2017(2) |
| P4.3 | Male | 2 | Recurrent oral ulcers, diarrhea | - | - | c.559+1G>A  splicing variants | Haploinsufficiency | BADRAN Y R, et al. 2017(2) |
| P4.4 | Female | 8 | Recurrent oral ulcers | - | - | c.559+1G>A  splicing variants | Haploinsufficiency | BADRAN Y R, et al. 2017(2) |
| P5 | Male | 5 | Autoimmune lymphoproliferative syndrome (pancytopenia, splenomegaly, lymphadenopathy, aseptic meningitis) | Splenectomy, glucocorticoids, mycophenolate mofetil (MMF), intravenous immunoglobulin (IVIG), rituximab | Improved | c.736C>T  p.R246* | Haploinsufficiency | COMRIE W A, et al. 2018(3) |
| P6.1 | Female | 1.5 | systemic lupus erythematosus (SLE) (facial rash, oral ulcers, polyarthritis, alopecia, fever) | Glucocorticoids, rituximab | Improved | c.256C>A  p.H86N | Dominant-negative | LAURA B, et al. 2020(4) |
| P6.2 | Female | 33 | SLE (polyarthritis, oral ulcers, pericarditis, lupus nephritis) | - | - | c.256C>A  p.H86N | Dominant-negative | LAURA B, et al. 2020(4) |
| P7 | Male | 9 | SLE (lupus rash, oral and limb skin ulcers, joint pain, lupus nephritis, pericarditis) | Hydroxychloroquine, glucocorticoids, MMF | Improved | c.985C>T  p.R329* | Dominant-negative | LAURA B, et al. 2020(4) |
| P8.1 | Male | 15 | Recurrent oral ulcers | - | Spontaneous remission | c.1459delC  p.H487Tfs*7 | Dominant-negative | ADEEB F, et al. 2021(5) |
| P8.2 | Female | 10 | Behçet’s disease (recurrent oral and genital ulcers, pyoderma) | Etanercept | Improved | c.1459delC  p.H487Tfs*7 | Dominant-negative | ADEEB F, et al. 2021(5) |
| P8.3 | Female | 15 | Behçet’s disease (recurrent oral and genital ulcers, pyoderma) | Etanercept | Improved | c.1459delC  p.H487Tfs*7 | Dominant-negative | ADEEB F, et al. 2021(5) |
| P8.4 | Female | 22 | Neuromyelitis optica (recurrent optic neuritis without transverse myelitis or brain involvement) | Rituximab | Improved | c.1459delC  p.H487Tfs*7 | Dominant-negative | ADEEB F, et al. 2021(5) |
| P8.5 | Female | 10 | Recurrent oral ulcers | - | - | c.1459delC  p.H487Tfs*7 | Dominant-negative | ADEEB F, et al. 2021(5) |
| P9.1 | Female | Infancy | Behçet’s disease (recurrent oral and genital ulcers, ocular hypertension, optic atrophy) | Glucocorticoids, colchicine | Improved | c.1044dupC  p.Y349Lfs*13 | Dominant-negative | LECERF K, et al. 2023(6) |
| P9.2 | Male | 2 | Behçet’s disease (recurrent oral and genital ulcers, scleritis, erythema nodosum, recurrent fever, intermittent diarrhea) | Glucocorticoids, colchicine | Improved | c.1044dupC  p.Y349Lfs*13 | Dominant-negative | LECERF K, et al. 2023(6) |
| P9.3 | Female | - | Behçet’s disease | - | - | c.1044dupC  p.Y349Lfs*13 | Dominant-negative | LECERF K, et al. 2023(6) |
| P9.4 | Female | 12 | Behçet’s disease (recurrent oral and genital ulcers, gastrointestinal mucosal ulcers) | Apremilast | Improved | c.1044dupC  p.Y349Lfs*13 | Dominant-negative | LECERF K, et al. 2023(6) |
| P9.5 | Male | 0 (1 month) | Behçet’s disease (recurrent oral and genital ulcers, recurrent conjunctivitis, urticaria-like rash, cervical lymphadenopathy, failure to thrive) | Anakinra, canakinumab | Improved | c.1044dupC  p.Y349Lfs*13 | Dominant-negative | LECERF K, et al. 2023(6) |
| P9.6 | Female | - | Behçet’s disease with Crohn’s disease | - | - | c.1044dupC  p.Y349Lfs*13 | Dominant-negative | LECERF K, et al. 2023(6) |
| P9.7 | Female | - | Behçet’s disease with Crohn’s disease | - | - | c.1044dupC  p.Y349Lfs*13 | Dominant-negative | LECERF K, et al. 2023(6) |
| P10.1 | Female | 8 | Behçet’s disease (recurrent headache, recurrent fever, recurrent oral and genital ulcers) | Glucocorticoids, colchicine, hydroxychloroquine | Improved | c.1153C>T  p.Q385* | Unknown | AN J W, et al. 2023(7) |
| P10.2 | Female | 15 | Recurrent oral and genital ulcers | Glucocorticoids | Improved | c.1153C>T  p.Q385* | Unknown | AN J W, et al. 2023(7) |
| P10.3 | Female | 29 | Recurrent oral and genital ulcers, rash, joint pain | Glucocorticoids, colchicine, etanercept | Improved | c.1153C>T  p.Q385* | Unknown | AN J W, et al. 2023(7) |
| P10.4 | Male | 9 | Recurrent oral ulcers | Colchicine | Improved | c.1153C>T  p.Q385* | Unknown | AN J W, et al. 2023(7) |
| P11.1 | Male | Childhood | Recurrent rash | - | - | c.1311_1312insA  p.E438Rfs*9 | Unknown | AN J W, et al. 2023(7) |
| P11.2 | Female | 2 | Behçet’s disease (recurrent oral and genital ulcers, rash, joint pain, headache, periodontitis) | Infliximab, adalimumab | Improved | c.1311_1312insA  p.E438Rfs*9 | Unknown | AN J W, et al. 2023(7) |
| P11.3 | Female | 3 | Behçet’s disease (recurrent oral and genital ulcers, recurrent fever, rash, joint pain) | Infliximab | Improved | c.1311_1312insA  p.E438Rfs*9 | Unknown | AN J W, et al. 2023(7) |
| P11.4 | Female | 0 (40 days) | Behçet’s disease (recurrent oral and genital ulcers, recurrent fever, rash, joint pain, headache, chronic gastroenteritis), recurrent infections | Adalimumab | Improved | c.1311_1312insA  p.E438Rfs*9 | Unknown | AN J W, et al. 2023(7) |
| P11.5 | Male | 5 | Behçet’s disease (recurrent oral and genital ulcers, rash, headache) | Adalimumab | Improved | c.1311_1312insA  p.E438Rfs*9 | Unknown | AN J W, et al. 2023(7) |
| P12.1 | Female | 10 | Chronic gastroenteritis | - | - | c.985C>T  p.R329* | Dominant-negative | AN J W, et al. 2023(7) |
| P12.2 | Female | 10 | SLE + Sjögren’s syndrome (recurrent fever, rash, joint pain, dry eyes) | Golimumab, adalimumab | Improved | c.985C>T  p.R329* | Dominant-negative | AN J W, et al. 2023(7) |
| P12.3 | Male | 9 | Recurrent fever, joint pain, chronic gastroenteritis | Etanercept | Improved | c.985C>T  p.R329* | Dominant-negative | AN J W, et al. 2023(7) |
| P13.1 | Female | 26 | Conjunctivitis | - | - | c.736C>T  p.R246* | Haploinsufficiency | AN J W, et al. 2023(7) |
| P13.2 | Male | 2 | Recurrent oral ulcers, abdominal pain | - | - | c.736C>T  p.R246* | Haploinsufficiency | AN J W, et al. 2023(7) |
| P13.3 | Male | 0 (Newborn) | Recurrent oral and genital ulcers, recurrent fever, rash, recurrent diarrhea, muscle weakness, recurrent infections | Glucocorticoids, etanercept | Improved | c.736C>T  p.R246* | Haploinsufficiency | AN J W, et al. 2023(7) |
| P14.1 | Male | 0 (1 month) | Recurrent fever, scoliosis, colitis | Infliximab | Improved | c.1165C>T  p.Q389* | Dominant-negative | UCHIDA T, et al. 2020(8) |
| P14.2 | Female | 13 | Juvenile idiopathic arthritis | - | - | c.1165C>T  p.Q389* | Dominant-negative | UCHIDA T, et al. 2020(8) |
| P15.1 | Male | 0 (1 month) | Recurrent fever, colonic ulcers, scoliosis | Infliximab | Improved | c.1165C>T  p.Q389* | Dominant-negative | MORIYA K, et al. 2023(9) |
| P15.2 | Female | Childhood | Recurrent oral ulcers, juvenile idiopathic arthritis | - | - | c.1165C>T  p.Q389* | Dominant-negative | MORIYA K, et al. 2023(9) |
| P16 | Male | 0 (Newborn) | Chronic immunologic thrombocytopenic purpura, autoimmune neutropenia, inflammatory bowel disease | Hematopoietic stem cell transplantation | Improved | c.985C>T  p.R329* | Dominant-negative | MORIYA K, et al. 2023(9) |
| P17 | Male | 2 | Recurrent fever, painful subcutaneous nodules, generalized myalgia | - | - | c.1416dup  p.E473fs | Dominant-negative | MORIYA K, et al. 2023(9) |
| P18 | Male | 0 (6 month) | Inflammatory bowel disease (recurrent fever, failure to thrive) | Adalimumab | Improved | c.1034-1G>A  splicing variants | Dominant-negative | MORIYA K, et al. 2023(9) |
| P19 | Male | 1.5 | Recurrent infections (bacterial and mycotic, generalized bullous lesions, conjunctivitis) | Glucocorticoids | Improved | c.1047T>A  p.Y349* | Dominant-negative | MORIYA K, et al. 2023(9) |

* GRCh37/hg19; NM_021975.4/ENST00000406246.8.

**References**

1. Frederiksen AL, Larsen MJ, Brusgaard K, Novack DV, Knudsen PJ, Schroder HD, et al. Neonatal High Bone Mass With First Mutation of the NF-kappaB Complex: Heterozygous De Novo Missense (p.Asp512Ser) RELA (Rela/p65). *J Bone Miner Res* (2016) 31(1): 163-72. doi:10.1002/jbmr.2590

2. Badran YR, Dedeoglu F, Leyva Castillo JM, Bainter W, Ohsumi TK, Bousvaros A, et al. Human *RELA* haploinsufficiency results in autosomal-dominant chronic mucocutaneous ulceration. *J Exp Med* (2017) 214(7): 1937-47. doi:10.1084/jem.20160724

3. Comrie WA, Faruqi AJ, Price S, Zhang Y, Rao VK, Su HC, et al. RELA haploinsufficiency in CD4 lymphoproliferative disease with autoimmune cytopenias. *J Allergy Clin Immunol* (2018) 141(4): 1507-10. doi:10.1016/j.jaci.2017.11.036

4. Laura B, Hicham L, Mathieu C, Nadia J, Marie-Claude S, Loïc C, et al. Heterozygous RELA mutations cause early-onset systemic lupus erythematosus by hijacking the NF-κB pathway towards transcriptional activation of type-I Interferon genes. *Biorxiv* (2020): 2020.04.27.046102. doi:10.1101/2020.04.27.046102

5. Adeeb F, Dorris ER, Morgan NE, Lawless D, Maqsood A, Ng WL, et al. A Novel RELA Truncating Mutation in a Familial Behçet’s Disease–like Mucocutaneous Ulcerative Condition. *Arthritis & Rheumatology (Hoboken, N.J.)* (2021) 73(3): 490-97. doi:10.1002/art.41531

6. Lecerf K, Koboldt DC, Kuehn HS, Jayaraman V, Lee K, Mihalic Mosher T, et al. Case report and review of the literature: immune dysregulation in a large familial cohort due to a novel pathogenic *RELA* variant. *Rheumatology (Oxford)* (2023) 62(1): 347-59. doi:10.1093/rheumatology/keac227

7. An JW, Pimpale-Chavan P, Stone DL, Bandeira M, Dedeoglu F, Lo J, et al. Case report: Novel variants in RELA associated with familial Behcet's-like disease. *Front Immunol* (2023) 14:1127085. doi:10.3389/fimmu.2023.1127085

8. Uchida T, Suzuki T, Kikuchi A, Kakuta F, Ishige T, Nakayama Y, et al. Comprehensive Targeted Sequencing Identifies Monogenic Disorders in Patients With Early‐onset Refractory Diarrhea. *J Pediatr Gastroenterol Nutr* (2020) 71(3): 333-39. doi:10.1097/MPG.0000000000002796

9. Moriya K, Nakano T, Honda Y, Tsumura M, Ogishi M, Sonoda M, et al. Human *RELA* dominant-negative mutations underlie type I interferonopathy with autoinflammation and autoimmunity. *J Exp Med* (2023) 220(9). doi:10.1084/jem.20212276
